# Belimumab in Lupus Nephritis: A Systematic Review and Meta-Analysis

**DOI:** 10.7759/cureus.20440

**Published:** 2021-12-15

**Authors:** Sanjeev Shrestha, Pravash Budhathoki, Yuvraj Adhikari, Anupama Marasini, Shakar Bhandari, Wasey Ali Yadullahi Mir, Dhan B Shrestha

**Affiliations:** 1 Department of Internal Medicine, Geisinger Health System, Danville, USA; 2 Department of Internal Medicine, BronxCare Health System, Bronx, USA; 3 Medicine, Nepalese Army Institute of Health Sciences, Kathmandu, NPL; 4 Department of Internal Medicine, Mount Sinai Hospital, Chicago, USA

**Keywords:** monoclonal antibody, meta-analysis, systemic lupus erythematosus, lupus nephritis, belimumab

## Abstract

Belimumab is a recombinant human IgG-1λ monoclonal antibody. It inhibits the B-cell activating factor (BAFF) and is approved for patients with systemic lupus erythematosus (SLE) older than five years with positive autoantibody. We aimed to evaluate the role of belimumab in the maintenance phase of treatment for lupus nephritis (LN). PubMed, PubMed Central (PMC), Cochrane Library, and Embase were searched using appropriate keywords. The screening of title and abstract was done in Covidence, followed by data extraction of the relevant studies based on inclusion criteria. Review manager (RevMan 5.4) was used for data analysis with random or fixed effects model based on heterogeneities. Two randomized controlled trials were included in the quantitative analysis. There were 1.71 times higher odds of complete renal response in the belimumab group than in the control group (odds ratio (OR), 1.71; 95% confidence interval (CI), 1.12-2.60; I-square (I^2^) ​​​​= 0%). Similarly, there was 34% lower odds for having no response among the belimumab group (OR, 0.66; 95% CI, 0.45-0.96; I^2 ^= 0%). No significant differences between the two groups were observed for the occurrence of treatment-related adverse events (TRAEs) (OR, 1.07; 95% CI, 0.74-1.56; I^2^ = 0%), treatment-related serious adverse events (OR, 0.54; 95% CI, 0.15-1.96; I^2^ = 68%), and treatment-related infections (OR, 0.65; 95% CI, 0.27-1.55; I^2^ = 21%).Therefore, belimumab and standard treatment were instrumental for beneficial renal response in patients with lupus nephritis and were not associated with increased odds of adverse effect compared with the standard treatment alone.

## Introduction and background

Systemic lupus erythematosus (SLE) is a chronic inflammatory disease with variable clinical manifestations. Lupus nephritis (LN) is one of the most common and serious manifestations of SLE. Lupus activity involving the kidney is characterized by nephrotic range proteinuria and hematuria with lupus stage-specific immunological findings. The symptoms of LN vary with stage; however, proteinuria, hypertension, and later, renal failure occur in advanced disease. LN of clinical relevance is diagnosed when the creatinine clearance decrease by 30%, with proteinuria of >500 mg/dL, and renal biopsy histological findings support active LN [1]. It occurs in nearly 50% of patients with SLE but is not the only cause of kidney injury in SLE [2]. LN treatment consists of an induction phase and a maintenance phase. In the induction phase, combination therapy with an immunosuppressive agent including steroids, cyclophosphamide (CYC), or mycophenolate mofetil (MMF) is employed to induce immune quiescence and reduce the ongoing renal inflammation [3]. Induction is intended for a complete renal response and to reduce early damage, preserving renal function in the long run. The maintenance phase is intended to avoid renal flares, minimizing glucocorticoid exposure and associated toxicity due to the use of immunosuppressants. The present treatment regimens for LN are associated with high drug-associated toxicity and low treatment efficacy and adherence [3]. In this context, belimumab has emerged as one of the emerging drugs for treating lupus nephritis. Belimumab is a recombinant IgG-1λ monoclonal antibody that acts by inhibiting B-cell activation and is approved for patients with SLE aged greater than five years with autoantibody activity [4]. Belimumab targets soluble B-cell activating factor (BAFF) and possibly helps prevent the development of autoreactive B-cells [3]. The most common side effects of belimumab are nausea, diarrhea, pyrexia, nasopharyngitis, etc. We conducted this study to assess the efficacy and safety of belimumab in patients with lupus nephritis.

## Review

Methods

We followed the guidelines of the Preferred Reporting Items for Systematic Reviews and Meta-Analyses (PRISMA) to conduct this meta-analysis [5].

Study protocol

An initial search and review of the literature were done on research questions. As a result, the protocol was published in Prospero (CRD420212340448) on February 10, 2021 [6].

Information sources

Electronic databases such as PubMed Central (PMC), PubMed, Cochrane Library, and Embase were searched for relevant articles with keywords such as “belimumab” and “lupus nephritis.”

Study records

Data Management

All the articles that met the keyword were uploaded into the Mendeley Reference Manager software, where first screening for duplicates was carried out, and those files were imported into Covidence to check for any duplicates and formal screening.

Selection Process

Two of our authors acted as independent reviewers (SB and YA) and screened the articles independently based on title and abstract. Then, any conflicts that arose during the screening were resolved by the third reviewer (AM). Two reviewers (YA and AM) did full-text reviews independently, and the third reviewer (SB) reviewed and resolved disputes in the Covidence screening software based on the preset inclusion and exclusion criteria.

All comparative studies (RCTs, cohort studies, case-control studies, cross-sectional studies, etc.) comparing belimumab with placebo and other standard care in lupus nephritis were included in our study. Editorials, commentaries, viewpoints, and studies with no proper data regarding efficacy, safety between belimumab with Placebo, and other standard care in lupus nephritis were excluded.

Data Collection Process

Necessary data were collected from selected studies using a predesigned Population Intervention Comparison and Outcome (PICO) formatted Microsoft Excel form and checked by all three reviewers for accuracy. The data extraction form included study details such as study ID, year of the study, study population characteristics (e.g., total number, age, sex, and other relevant disease-specific parameters such as comorbidities), intervention (dose and duration of the study drug), comparator (placebo or standard care), and outcome (changes in base value, adverse effect, and mortality).

Outcome

The primary outcomes were efficacy in terms of good renal response and safety of belimumab in lupus nephritis.

Risk of bias in individual studies

**Figure 1:**
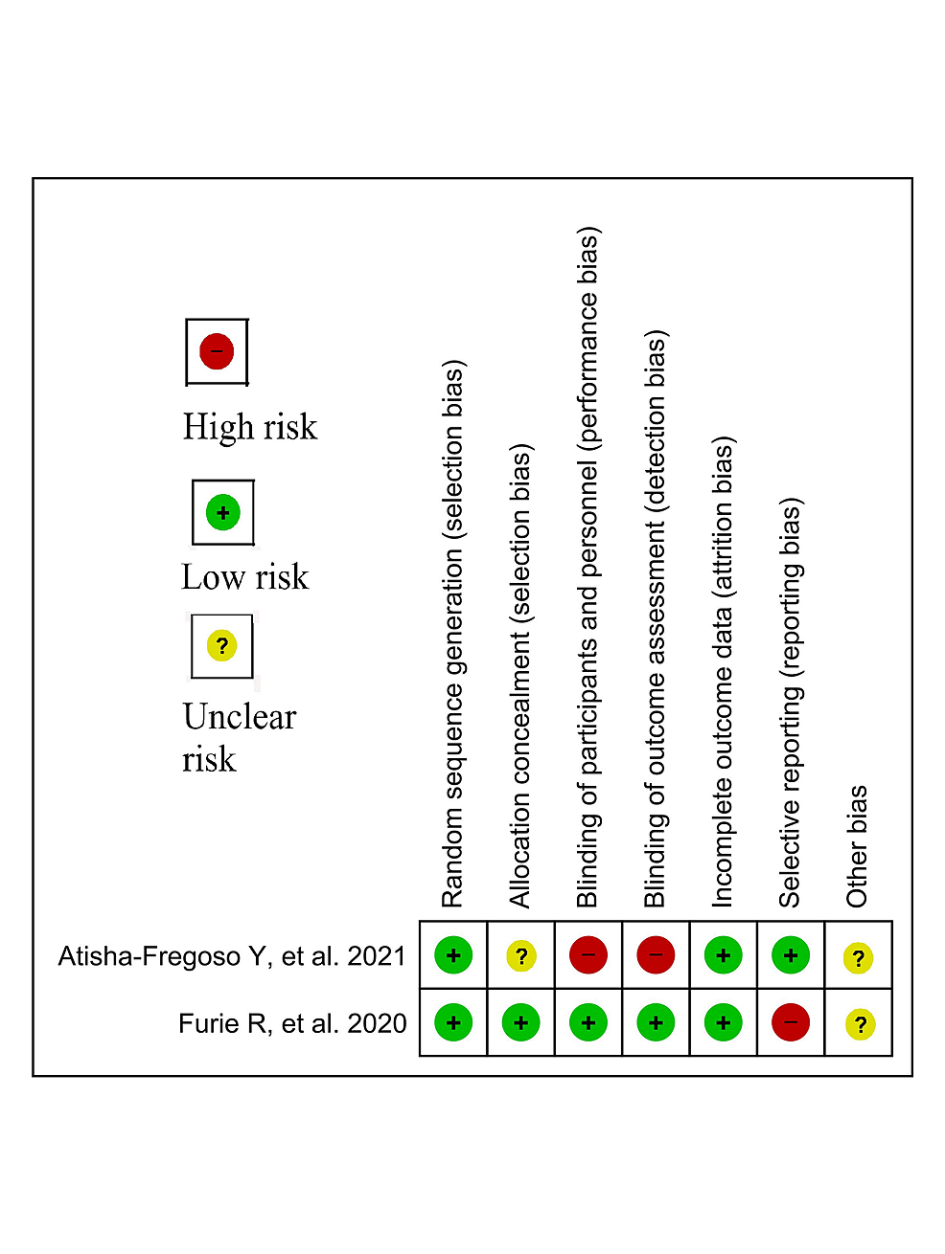
Risk-of-bias summary of randomized controlled trials

The individual articles were evaluated using Cochrane risk-of-bias tool for randomized trials (RoB 2.0) for their methodological quality (Figure 1) [7].

Data synthesis****

RevMan 5.4 was used for the analysis of the extracted data. Based on the heterogeneities across the studies, a random/fixed effects model was used.

Assessment of heterogeneity

Assessment of heterogeneity was done using I-square (I^2^) test based on the Cochrane Handbook for Systematic review [8].

Sensitivity analysis

Sensitivity analysis was not performed due to the paucity of studies regarding the use of belimumab in lupus nephritis.

Result

**Figure 2:**
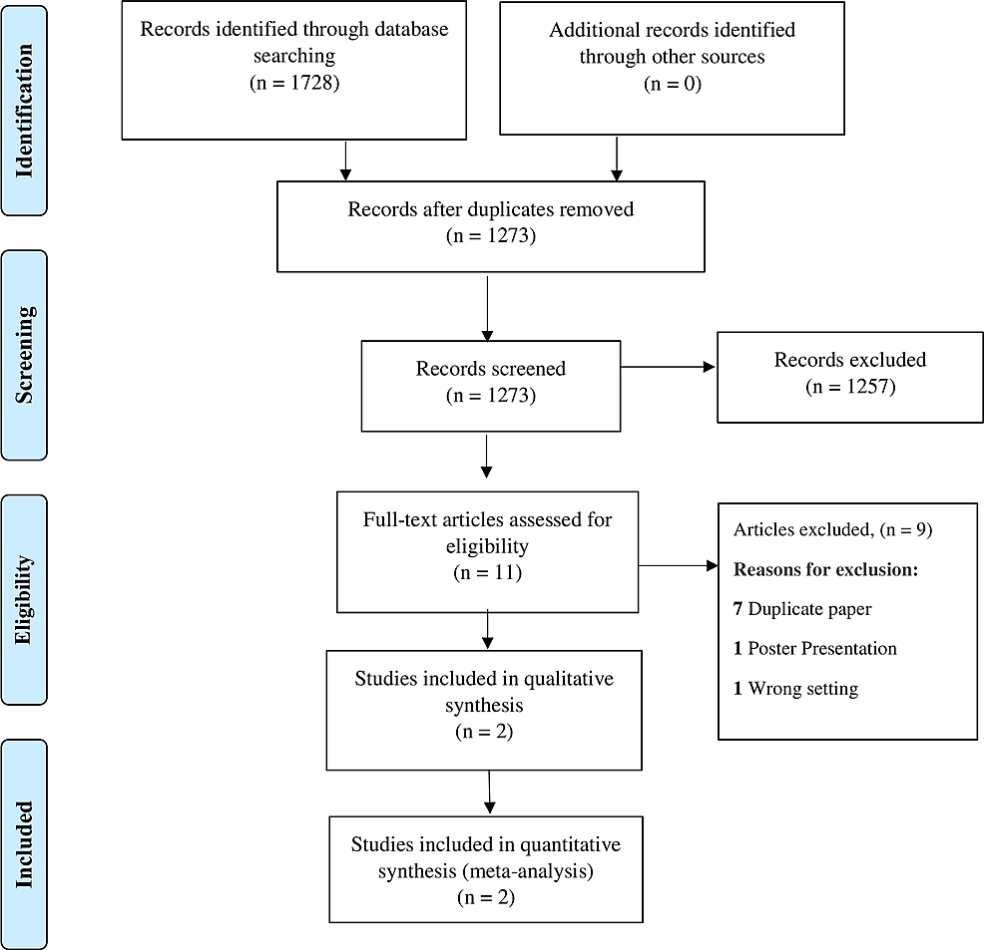
PRISMA flow diagram

A total of 1728 studies were identified after thorough database searching, and duplicates were removed. The titles and abstracts of 1273 studies were screened, and 1257 studies were excluded. In addition, 11 studies were assessed for full-text eligibility. Nine studies were excluded for definite reasons, and two studies were included in the qualitative and quantitative analysis (Tables 1, 2). The following information is presented on the PRISMA flow diagram (Figure 2).

**Table 1:** Baseline details of the included studies ANAs: antinuclear antibodies; DNA: deoxyribonucleic acid; SLE: systemic lupus erythematosus; MMF: mycophenolate mofetil; CYC: cyclophosphamide; LN: lupus nephritis; ACR: American College of Rheumatology; eGFR: estimated glomerular filtration rate; SLICC: Systemic Lupus International Collaborating Clinics; ISNRPS: International Society of Nephrology and Renal Pathology Society

ID	Country	Inclusion criteria	Exclusion criteria
Atisha-Fregoso et al., 2021 [3]	United States	1. Age > 18 years, diagnosis fulfilling the ACR or SLICC criteria for SLE 2. ANAs and/or anti-double-stranded DNA (anti-dsDNA) antibodies positive at the time of screening 3. Recurrent or refractory LN treated with either CYC or MMF previously	Already a case treated with rituximab or another B-cell biologic therapy within the prior 12 months
Furie et al., 2020 [4]	Multinational (21 countries, 107 sites)	1. Age ≥ 18 years; autoantibody-positive 2. SLE (ANA titers ≥ 1:80, anti-dsDNA antibodies, or both); urinary protein/creatinine ≥ 1 and biopsy-proven LN ISNRPS class III (focal lupus nephritis) or IV (diffuse lupus nephritis) with or without coexisting class V (membranous lupus nephritis) or pure class V lupus nephritis within six months before or during screening 3. Patients with active lesions or active and chronic lesions in biopsy	Dialysis within one year, eGFR < 30 mL/minute/1.73 m^2^ of body surface area; previous failure with either CYC or MMF induction; induction with CYP within three months before the trial; and B-cell–targeted therapy (including belimumab) one year before randomization

**Table 2:** Qualitative synthesis of the included studies T: treatment; TRAEs: treatment-emergent adverse events; C: control; N: total number of participants; SD: standard deviation; CYC: cyclophosphamide; eGFR: estimated glomerular filtration rate; IV: intravenous; AEs: adverse events

ID	Study design	Population	Intervention	Comparator	Primary outcome	Secondary outcomes	Other outcomes	
Atisha-Fregoso et al., 2021 [3]	Phase II multicenter, randomized, controlled, open-label trial	N = 43 (T = 21, C = 22)	Methylprednisolone 100 mg, rituximab 1000 mg, and CYC 750 mg intravenously (IV) at weeks 0 and 2 through week 4; weekly belimumab infusions at a dose of 10 mg/kg at weeks 4, 6, and 8 and every four weeks thereafter through week 48	Methylprednisolone 100 mg, rituximab 1000 mg, and 750 mg IV at weeks 0 and 2	Infectious TRAEs ≥grade 3 (week 48): T = 2/21, C = 5/22 Infectious AEs: T = 3, C = 7	Infectious TRAEs ≥grade 3: T = 2/21, C = 6/22	Deaths: T = 0, C= 0	
		Male: N = 6 (T = 2, C = 4)					Renal response at week 24	
						Total AEs: T = 5, C = 10		
		SCr, mean ± SD, mg/dL: (T = 1.02 ± 0.41, C = 1.04 ± 0.47)				TRAEs ≥grade 2: T = 21/21, C = 22/22		
							Complete: T = 5/21, C = 5/22	
						Serious TRAEs: T = 4/21, C = 11/22		
		eGFR, mean ± SD, mL/minute/1.73 m^2^: (T = 92.7 ± 36.0, C = 89.1 ± 33.9)					Partial: T = 5/21, C = 4/22	
							Nonresponse: T = 8/21, C = 8/22	
		B-cell count, median number of cells/μL: (T = 105.5, C = 143)					Withdrawal: T = 3/21, C = 5/22	
							Week 48 complete: T = 8/21, C = 7/22	
							Partial: T = 3/21, C = 2/22	
							Nonresponse: T = 3/21, C = 0/22	
							Withdrawal: T = 7/21, C = 13/22	
							Week 96 (C: n = 21) Complete: T = 5/21, C = 4/21	
							Partial: T = 1/21, C = 2/21	
							Nonresponse: T = 1/21, C = 0/21	
							Withdrawal: T = 14/21, C = 15/21	
Furie et al., 2020 [4]	Randomized, double-blind, placebo-controlled trial	N = 446 (T = 223, C = 223)	IV belimumab on days 1 (baseline), 15, and 29 and every 28 days thereafter to week 100 Standard induction therapy consisted of IV CYP (500 mg every two weeks (±3 days) for six infusions) or MMF (target dose, 3 g per day)	Placebo with standard therapy was given as a comparator	Renal response at week 104: T = 96/223 (43), C = 72/223 (32)	Complete response at week 104: T = 67/223 (30), C = 44/223 (20)	AEs: T = 214/224, C = 211/224	
		Female (N): (T = 197, C = 196)				Partial response: T = 39/223 (18), C = 38/223 (17)	TRAEs = T = 123/224, C = 119/224	
		Age: (T = 33.7 ± 10.7, C = 33.1 ± 10.6)				No response: T = 117/223 (52), C = 141/223 (63)		
							Treatment-related serious AEs: T = 23/224 (10), C = 25/224 (11)	
						Primary efficacy renal response at week 52: T = 104/223 (47), C = 79/223 (35)		
		Urinary protein to creatinine ratio: (T = 3.2 ± 2.7, C = 3.5 ± 3.6)					Serious infection and infestation: T = 15/224 (7), C = 18/224 (8)	
							AEs resulting to the discontinuation of trial: T = 29/223 (13), C = 29/223 (13)	
		eGFR, mL/minute/1.73 m^2^,(T = 100 ± 37.7, C = 101 ± 42.7)						
							Fatal AEs during the trial intervention: T = 4/223 (2), C = 3/223 (1)	

Efficacy of Belimumab in Lupus Nephritis

**Figure 3:**
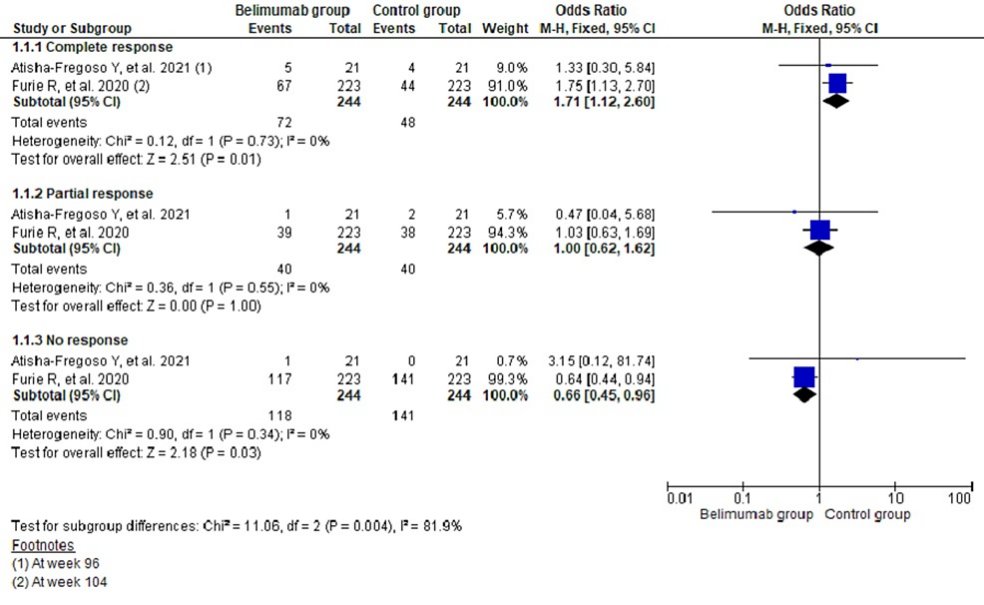
Forest plot showing renal response among the two groups of treatment recipient patients with lupus nephritis using fixed effects model CI: confidence interval

The pooling data of two studies (n = 488) showed that there were 1.71 times higher odds of complete renal response in the belimumab group than in the control group (odds ratio (OR), 1.71; 95% confidence interval (CI), 1.12-2.60; I^2^ = 0%). Similarly, there was 34% lower odds for having no response among the belimumab group (OR, 0.66; 95% CI, 0.45-0.96; I^2^ = 0%). However, there was no difference between the two groups regarding partial renal response (OR, 1.00; 95% CI, 0.62-1.62; I^2^ = 0%) (Figure 3).

Safety of Belimumab in Lupus Nephritis

**Figure 4:**
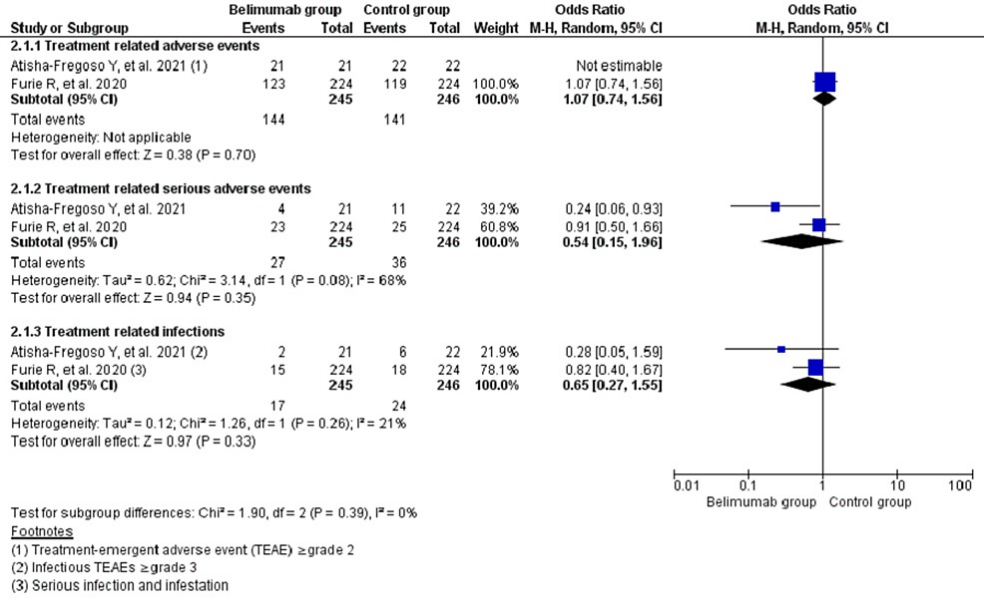
Forest plot showing treatment-related adverse events among the two groups of treatment recipient patients with lupus nephritis using a random effects model CI: confidence interval

The pooling data on adverse events during treatment using random effects model showed no significant differences between two groups for the occurrence of treatment-related adverse events (TRAEs) (OR, 1.07; 95% CI, 0.74-1.56; I^2^ = 0%), treatment-related serious adverse events (OR, 0.54; 95% CI, 0.15-1.96; I^2^ = 68%), and treatment-related infections (OR, 0.65; 95% CI, 0.27-1.55; I^2^ = 21%) (Figure 4).

Discussion

The meta-analysis showed higher odds of renal response in patients receiving belimumab compared with patients receiving standard of treatment and no significant difference in adverse effect between belimumab and standard treatment. These findings are significant because higher risks of relapse and poor term outcomes are typically observed in lupus nephritis [9-11]. The common findings in patients with lupus nephritis are increased production of BAFF in the kidney, leading to increased levels of BAFF in the blood [12-14]. To manage lupus nephritis, it is imperative to neutralize the B-cell activating factor to decrease B-cell function, autoantibody production, and suppression of lymphoid structure formation in the kidney. Belimumab has been shown to produce partial depletion of B-cells and cause low circulating levels of BAFF [15]. This might explain better renal response seen in patients with lupus nephritis who received belimumab in addition to usual standard medications compared with standard of treatment alone. The prevention of renal flare is instrumental in the prevention of worse outcomes seen with lupus nephritis. The odds for the adverse event were similar in a patient receiving belimumab in addition to standard treatment compared with standard treatment alone. A similar finding was seen in the Rituximab and Belimumab for Lupus Nephritis (CALIBRATE) trial in which belimumab was found to be a safe treatment in lupus nephritis [3]. The safety of belimumab in the treatment of SLE has already been well established, and a prior meta-analysis done by Wei et al. found no significant difference in the adverse event of belimumab in SLE [16].

Our meta-analysis is the first meta-analysis to evaluate the efficacy and safety of belimumab in lupus nephritis performed after a comprehensive search of various databases. However, our study has a few limitations. There was a lack of an adequate number of studies in our analysis. The included studies had their inherent limitations, such as low enrollment of Black patients and patients receiving cyclophosphamide and azathioprine, as well as lack of patient-reported outcomes in the Belimumab in Subjects With Systemic Lupus Erythematosus (BLISS) trial [4]. Similarly, the CALIBRATE trial was underpowered and included patients with only refractory or recurrent lupus nephritis [3].

## Conclusions

Belimumab, a monoclonal antibody, is a newer treatment option for SLE in reducing the side effect of immunomodulatory drugs. Belimumab has shown promising results in patients with lupus. Its addition to standard treatment showed an improved renal response in patients with lupus nephritis and was not associated with increased odds of adverse effects compared with the standard treatment alone. Due to the paucity of studies among patients with lupus nephritis, further study in this subgroup of patients with lupus is recommended to affirm these initial promising findings.

## Appendices

Belimumab and lupus nephritis electronic search details

PubMed

Keywords: “belimumab” and “lupus nephritis”

Hits: 124

Link: https://pubmed.ncbi.nlm.nih.gov/?term=belimumab+and+lupus+nephritis

PubMed Central

Keywords: “belimumab” and “lupus nephritis”

Hits: 787

Link: https://www.ncbi.nlm.nih.gov/pmc/?term=%22belimumab%22+and+%22lupus+nephritis%22

Cochrane Library

Keywords: “belimumab” in All Text AND “lupus nephritis” in All Text 

Hits: 20

Link: https://www.cochranelibrary.com/advanced-search

Embase

Keywords: (“belimumab”/exp OR belimumab) AND (“lupus nephritis”/exp OR “lupus nephritis”)

Hits: 549

Link: https://www.embase.com/#advancedSearch/resultspage/history.2/page.1/25.items/orderby.date/source
